# Musculoskeletal disorders as underlying cause of death in 58 countries, 1986–2011: trend analysis of WHO mortality database

**DOI:** 10.1186/s12891-017-1428-1

**Published:** 2017-02-02

**Authors:** Aliasghar A. Kiadaliri, Anthony D. Woolf, Martin Englund

**Affiliations:** 1 Department of Clinical Sciences Lund, Lund University, Faculty of Medicine, Orthopaedics, Skåne University Hospital, Clinical Epidemiology Unit, Klinikgatan 22, SE-221 85 Lund, Sweden; 2 Bone and Joint Research Group, The Knowledge Spa, Royal Cornwall Hospital, Truro, UK; 3 Clinical Epidemiology Research and Training Unit, Boston University School of Medicine, Boston, MA USA

## Abstract

**Background:**

Due to low mortality rate of musculoskeletal disorders (MSK) less attention has been paid to MSK as underlying cause of death in the general population. The aim was to examine trend in MSK as underlying cause of death in 58 countries across globe during 1986–2011.

**Methods:**

Data on mortality were collected from the WHO mortality database and population data were obtained from the United Nations. Annual sex-specific age-standardized mortality rates (ASMR) were calculated by means of direct standardization using the WHO world standard population. We applied joinpoint regression analysis for trend analysis. Between-country disparities were examined using between-country variance and Gini coefficient. The changes in number of MSK deaths between 1986 and 2011 were decomposed using two counterfactual scenarios.

**Results:**

The number of MSK deaths increased by 67% between 1986 and 2011 mainly due to population aging. The mean ASMR changed from 17.2 and 26.6 per million in 1986 to 18.1 and 25.1 in 2011 among men and women, respectively (median: 7.3% increase in men and 9.0% reduction in women). Declines in ASMR of 25% or more were observed for men (women) in 13 (19) countries, while corresponding increases were seen for men (women) in 25 (14) countries. In both sexes, ASMR declined during 1986–1997, then increased during 1997–2001 and again declined over 2001–2011. Despite decline over time, there were substantial between-country disparities in MSK mortality and its temporal trend.

**Conclusions:**

We found substantial variations in MSK mortality and its trends between countries, regions and also between sex and age groups. Promoted awareness and better management of MSK might partly explain reduction in MSK mortality, but variations across countries warrant further investigations.

**Electronic supplementary material:**

The online version of this article (doi:10.1186/s12891-017-1428-1) contains supplementary material, which is available to authorized users.

## Background

Musculoskeletal disorders (MSK) cover a wide range of disorders affecting joints, bones, muscles and soft tissues. Many MSK are recurrent or lifelong disorders [1]. The main consequences of MSK are typically long term pain, physical disability, loss of independence, reduced social interaction, and a decline in quality of life [2]. Globally, 18.5% of years lived with disability was attributed to MSK in 2015 (68% increase from 1990) [3]. When taking into account both death and disability, all MSK combined accounted for 6.0% of the total global disability-adjusted life years [4]. Population growth, ageing, obesity, increased sedentary lifestyles, and work-related issues imply that the number of people suffering from MSK (and, thus the burden from MSK) will increase dramatically worldwide over the coming decades [5].

Increased risk of mortality may be another consequence of MSK, even though for the majority of MSK the mortality rate is low. Despite this, to have an accurate estimation of burden of MSK, mortality associated with MSK must also be measured [1]. Previous studies reported higher risk of mortality among people with some MSK including rheumatoid arthritis (RA) and osteoarthritis (OA) compared with the general population possibly due to increased risk of cardiovascular disease and infection [6–10]. However, due to low mortality rate less attention has been paid to MSK as underlying cause of death in the general population.

To our best knowledge, only a recent study investigated temporal trend in mortality with MSK as underlying cause in Sweden [11]. MSK mortality has been influenced by the emergence of new advances in MSK management including introduction of biological agents for RA. For example, previous studies reported that biological agents are associated with increased risk for serious infectious events and declined risk of cardiovascular events [12–14]. Considering this new advances and also scarcity of study on MSK mortality, an update on the trends in MSK mortality is needed. In addition, we quantified the magnitude of the absolute and relative between-country disparities in MSK mortality which has important policy implications. Furthermore, we decomposed changes in number of MSK deaths into demographic and epidemiologic changes which are important in health systems policy making. The aim of the current study was to investigate the trends in MSK mortality rates and associated between-country disparities using data from the World Health Organization (WHO) mortality database.

## Methods

### Data sources

Annual data on all-cause mortality and mortality due to MSK as underlying cause of death during 1986–2011 were obtained from the WHO mortality database (http://www.who.int/healthinfo/mortality_data/en/, accessed April 2016). This database provides annual data on underlying cause of death by age, sex, and cause of death as submitted by national death registration systems. International Classification of Diseases (ICD) codes were used to extract data on MSK mortality (ICD-8 and ICD-9 codes 710–740, and ICD-10 codes M00-M99). Several countries used different codes for MSK deaths which was taken into account in extracting the data. Due to low number of MSK deaths, we only included countries with more than one million population in 2010. In total, 58 countries (United Kingdom included as three countries: England & Wales, Northern Ireland, and Scotland) had required data for our analysis and were included. The application of ICD revisions varied across countries during the study period (Additional file 1: Table S1).

While there were no missing data for 49 countries, in 9 countries missing data points ranged from one in Kazakhstan and Dominican Republic to 6 in Panama resulting in a total of 42 missing values out of 3016 sex-country-year data points. Missing values were imputed using multiple imputation (10 imputations) applying Poisson regression model adjusted for year, sex, age group, and ICD revision with population as exposure (number of deaths was used as dependent variable). Population data by sex and age were obtained from the United Nations Population Prospects database (http://esa.un.org/unpd/wpp/). Moreover, we grouped these countries in 10 regions according to the United Nations Statistics Division (http://unstats.un.org/unsd/methods/m49/m49regin.htm): Asia (*n* = 7), Eastern Europe (*n* = 8), Northern Europe (*n* = 11), Southern Europe (*n* = 6), Western Europe (*n* = 6), Caribbean (*n* = 4), Central America (*n* = 4), South America (*n* = 8), North America (*n* = 2), and Oceania (*n* = 2).

### Trend analysis

We computed age-standardized mortality rates per million population by means of direct standardization using the WHO Reference Population [15]. Age-standardized rates per million population were calculated for each country/region and year for each sex. We also computed women to men age-standardized rate ratio and its 95% confidence interval (CI). The percent change was calculated as the difference between the rate of 1986 and 2011 divided by the rate of 1986.

Temporal trends in age-standardized mortality rate were analyzed by joinpoint regression using the Joinpoint Regression Program version 4.2.0.2 from the Surveillance Research Program of the US National Cancer Institute (http://surveillance.cancer.gov/joinpoint). Joinpoint regression identifies points with a significant change in trend (“joinpoints”) and determined linear trends between joinpoints. In the software a series of permutation tests is applied to compute the number of joinpoints to best fit the data [16]. For each joinpoint an annual percentage change (APC) is estimated by fitting a regression line to the natural logarithm of the age-standardized rates, using calendar year as a predictor. The average annual percent change (AAPC) as the weighted average of APCs was computed to provide a summary measure of the trend for the whole time period [17]. Since recent trends are possibly the best predictor of mortality rates in coming years and also to avoid biases caused by differences in ICD revision, we additionally computed the percentage changes and AAPCs for period 2001–2011.

### Between-country disparity

We used between-country variance (BCV) to examine the trend in absolute between-country disparity in MSK mortality. The BCV was calculated using following formula [18].

$$ B C V={\displaystyle \sum_{j=1}^J}{p}_j{\left({y}_j-\mu \right)}^2 $$

Where p_j_ is country j’s proportion of the total population, y is country j’s age-standardized MSK mortality rate, and μ is the pooled age-standardized MSK mortality rate of all countries.

Gini coefficient was used for examining changes in relative between-country disparities. Gini coefficient is a commonly used disparity measure [18] and is based on the Lorenz curve which plots the cumulative share of population ranked by health variable, in an increasing order, against the cumulative share of health variable. The Gini coefficient is equal to twice the area between the Lorenz curve and diagonal. Its value ranges from 0 (perfect equality) to one (maximum possible inequality).

### Decomposition analysis

We decomposed the drivers of changes in the number of MSK deaths between 1986 and 2011 into three components using two counterfactual scenarios [19]: 1) population growth scenario using the number of population in 2011 and the age-sex structure and MSK death rates in 1986, 2) population growth and aging scenario using the number of population and the age-sex structure in 2011 and MSK death rates in 1986. Difference between the actual number of deaths in 1986 and those estimated from the population growth scenario is the change due to *population growth*. The difference between the population growth scenario and population growth and aging scenario is the change due to *population aging*. The difference between the actual number of deaths in 2011 and the population growth and aging scenario is the change due to *epidemiologic changes*. The epidemiologic changes are changes in the age-, sex-, and cause-specific rates of death and include all changes in mortality that cannot be explained by population growth and aging. The actual change in the number of deaths between 1986 and 2011 is equal to the net change in these three components.

## Results

### Number of deaths and proportion of all causes deaths

In total, 192 666 069 men and 177 582 994 women died in 58 countries during 1986–2011, of these 419 848 men and 956 011 women died with MSK as underlying cause. About 70 and 75% of all MSK deaths were observed in men and women aged 65 years and older, respectively. On average, MSK deaths constituted 0.22% (ranging from 0.02% in Romania to 0.48% in Spain) and 0.54% (ranging from 0.05% in Romania to 1.28% in Spain) of all-cause deaths among men and women, respectively. Among men, MSK deaths increased from 11861 deaths in 1986 (0.18% of all-cause deaths) to 22380 deaths in 2011 (0.28% of all-cause deaths, Additional file 2: Figure S1), representing an increase of 89% (ranging from 85.3% reduction in Romania to 2600% increase in Greece). Among women, MSK deaths increased from 28 272 in 1986 (0.46% of all-cause deaths) to 44 652 in 2011 (0.61% of all-cause deaths), corresponding to an increase of 58% (ranging from 71.9% reduction in Romania to 552.9% increase in Greece). Across regions, the highest reduction in the number of MSK deaths between 1986 and 2011 was seen in Eastern Europe for men and in Northern Europe for women. South America had the highest increases in the number of MSK deaths for both sexes.

### Age-standardized mortality rate

**Fig. 1:**
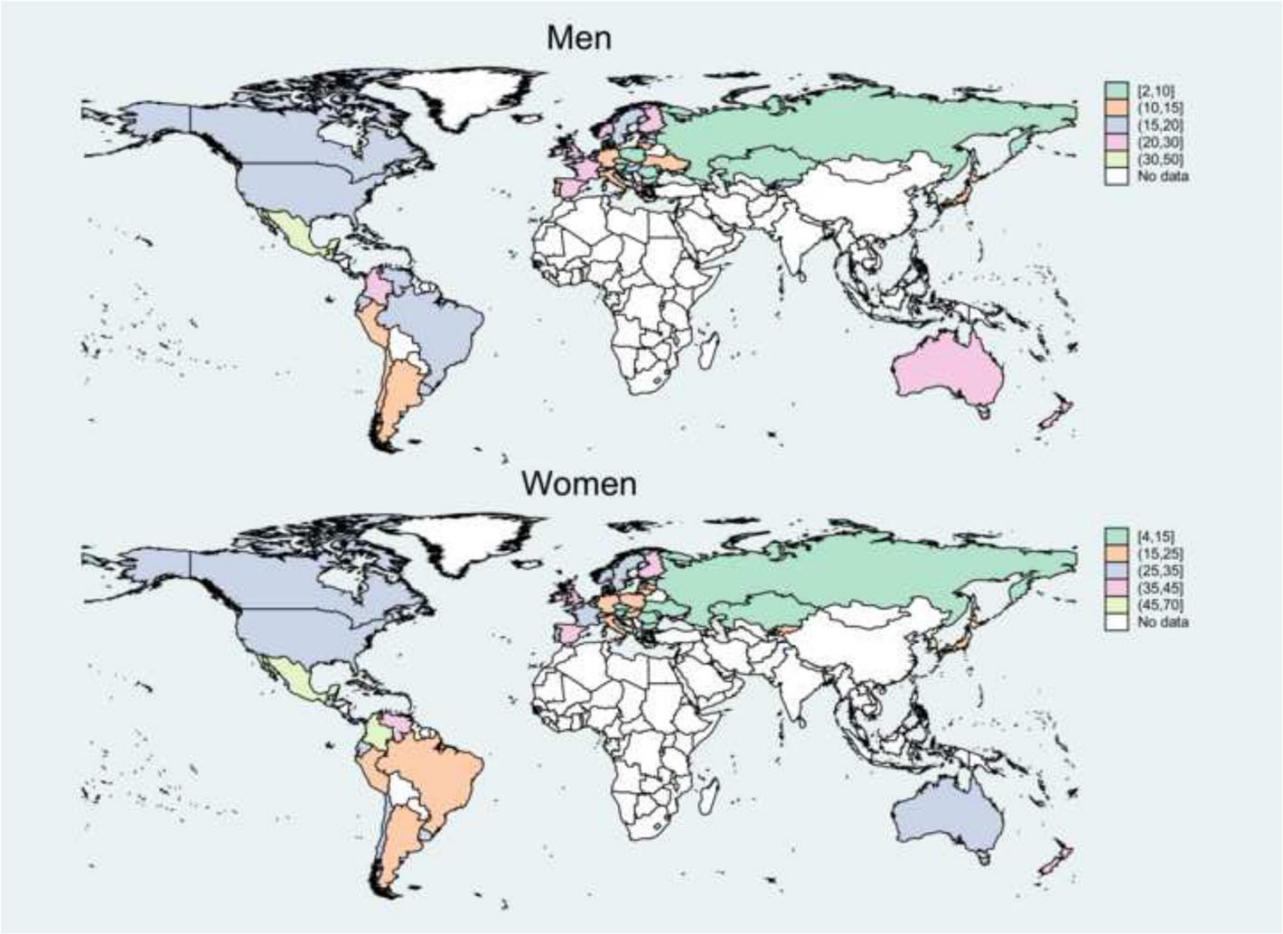
The mean age-standardized musculoskeletal disorders mortality rates per million person-years, 1986–2011

The pooled age-standardized MSK mortality rates were 17.7 and 26.5 deaths per million person-years among men and women, respectively (women to men rate ratio of 1.5). The mean MSK mortality rates ranged from 2.6 deaths per million person-years in Romania to 45.3 in Trinidad and Tobago for men, and from 4.4 in Bulgaria to 68.9 in Trinidad and Tobago for women (Fig. 1). In all countries but Guatemala and Greece, women had statistically significantly higher MSK mortality rate than men (Additional file 1: Table S2). Among regions, the highest and lowest MSK mortality rates for both sexes were observed in Central America and Eastern Europe, respectively (Additional file 3: Figure S2).

**Fig. 2:**
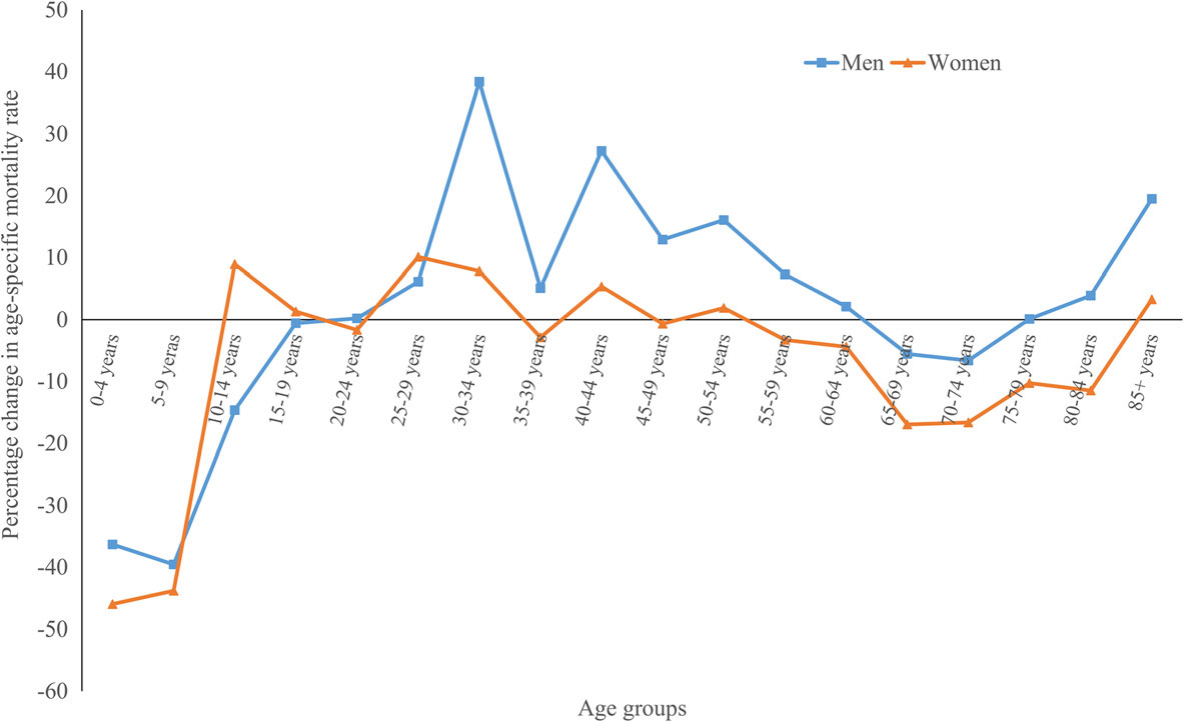
Percent change in age-specific musculoskeletal mortality rates per million person-years between 1986 and 2011

The mean MSK mortality rate increased from 17.2 deaths per million person-years in 1986 to 18.1 in 2011 for men, representing a 5.3% increase (ranging from an 86% reduction in Panama to a 1325% increase in Greece with a median of 7.3% increase, Table 1). Mortality reductions of 25% or more were observed in 13 countries, while increases of 25% or more were seen in 25 countries. The absolute change in MSK mortality rate ranged from about 50 less deaths per million person-years in Guatemala to about 19.5 more deaths in Greece between 1986 and 2011. Across regions, the highest MSK mortality decline was observed in Central America (28% reduction) and the highest increase in Caribbean (58% increase). Between 1986 and 2011, age-specific MSK mortality rate declined in men 0–19 years and 65–74 years and increased in all other age groups (Fig. 2).

**Table 1:** Changes in musculoskeletal disorders age-standardized mortality rate per million person-years 1986–2011, stratified by sex

Country/region	Men							Women						
	Mortality rate			% ∆		Annual change, %		Mortality rate			% ∆		Annual change, %	
	1986	2001	2011	1986 to 2011	2001 to 2011	1986 − 2011	2001 − 2011	1986	2001	2011	1986 to 2011	2001 to 2011	1986 − 2011	2001 − 2011
All countries (*n* = 58)	17.2	18.9	18.1	5.3	−3.9	0.2	−0.3*	26.6	27.8	25.1	−5.6	−9.8	−0.3	−1.2*
Asia (*n* = 7)	14.7	16.9	16.0	8.9	−5.1	0.5	−0.1	26.6	24.7	19.0	−28.6	−23.0	−1.3*	−2.9*
Hong Kong SAR	2.6	16.0	10.6	309.0	−33.8	7.8*	−2.5	19.9	15.5	11.9	−39.9	−23.3	−1.1	−2.3*
Israel	8.0	18.2	26.1	224.3	43.1	5.3*	0.8	17.7	20.0	27.1	53.1	35.8	2.0*	0.9
Japan	12.8	14.2	14.9	16.0	4.4	0.7	0.8	28.2	20.5	17.3	−38.7	−15.9	−2.1*	−1.8*
Kazakhstan	12.5	10.4	7.4	−41.1	−29.2	−2.6*	−3.7*	13.8	12.4	15.7	14.0	26.7	0.3	1.9
Kyrgyzstan	7.2	14.0	23.6	228.0	68.8	3.3	4.2	11.9	15.1	32.7	173.8	116.5	4.0*	3.8
Republic of Korea	31.8	39.7	24.3	−23.7	−38.8	−0.3	−4.1*	26.5	60.7	27.3	2.9	−55.1	1.1	−8.7*
Singapore	8.9	5.0	7.5	−15.4	49.5	−0.7	2.5	27.7	12.4	6.5	−76.5	−47.2	−5.1*	−3.2
Eastern Europe (*n* = 8)	9.4	8.9	7.2	−23.4	−18.6	−1.0*	−1.9*	14.2	13.5	10.5	−26.5	−22.8	−1.3*	−2.7*
Bulgaria	2.4	4.2	5.4	121.2	28.0	1.3	0.7	3.2	2.3	5.6	74.7	143.2	1.0	5.8
Czech Republic	5.3	2.6	8.2	54.4	214.7	1.1	15.0*	8.5	3.4	7.8	−9.0	126.9	−1.3	10.1*
Hungary	13.4	14.0	19.3	43.6	37.6	1.9*	4.2*	27.3	21.8	25.1	−8.1	15.0	−0.1	2.0
Poland	13.2	6.3	5.1	−61.5	−20.0	−3.8*	−2.4	25.9	14.3	11.5	−55.7	−19.7	−3.5*	−3.1*
Republic of Moldova	12.8	8.4	8.5	−33.8	1.2	−1.9*	−0.2	12.4	12.9	5.5	−55.2	−57.0	−3.6*	−9.9*
Romania	5.7	1.3	0.9	−84.0	−27.3	−6.7*	−4.4	9.5	2.5	2.3	−75.6	−7.9	−6.0*	−1.2
Russian Federation	9.6	10.2	7.4	−23.3	−27.6	−1.1*	−2.8*	13.2	14.9	10.6	−19.8	−28.9	−0.8	−3.2*
Ukraine	8.6	11.6	8.6	−0.8	−26.2	0.1	−2.8*	12.3	16.1	11.0	−10.8	−31.6	−0.5	−3.6*
Northern Europe (*n* = 11)	30.6	26.4	23.6	−23.0	−10.6	−1.0	−1.3*	51.6	39.2	30.4	−41.1	−22.5	−2.1*	−2.8*
Denmark	13.1	20.9	23.4	78.9	12.1	2.5	1.0	20.0	41.3	25.7	28.2	−37.9	0.8	−4.6*
Estonia	13.9	14.2	13.6	−2.3	−4.3	0.0	−4.1	24.6	33.0	15.8	−35.8	−52.2	−1.2	−6.3*
Finland	21.6	23.4	13.4	−38.0	−42.9	−2.0*	−4.6*	45.4	31.5	21.3	−53.1	−32.4	−2.7*	−4.7*
Ireland	32.7	24.4	22.4	−31.3	−8.1	−1.8*	−1.3	37.7	34.1	28.2	−25.2	−17.1	−1.7	−1.0
Latvia	13.5	21.0	16.5	22.2	−21.3	1.9*	−1.1	23.6	18.5	20.2	−14.5	9.5	−1.2*	−1.4
Lithuania	12.1	14.3	9.8	−19.1	−31.9	0.6	−1.0	28.1	18.2	19.3	−31.4	6.2	−1.7*	−1.5
Norway	23.5	22.1	22.0	−6.1	−0.5	−0.1	−1.1	35.6	32.9	24.9	−29.9	−24.1	−1.8*	−3.6*
Sweden	13.6	15.5	17.4	28.7	12.4	−0.1	−0.1	24.5	28.3	20.1	−18.2	−29.0	−0.7	−2.0*
England & Wales	41.0	30.7	26.7	−35.0	−13.2	−1.9	−1.5*	65.9	44.2	35.4	−46.3	−20.1	−2.5*	−2.7*
Northern Ireland	11.8	20.7	23.1	95.8	11.3	3.1	1.0	21.5	41.5	33.7	56.6	−18.7	0.7	−1.7
Scotland	19.3	22.6	24.2	25.2	7.2	0.8*	−0.2	42.0	40.3	35.5	−15.4	−11.9	−0.5	−1.8*
Southern Europe (*n* = 6)	16.6	16.8	17.0	2.1	0.9	0.2	−0.8	25.0	24.5	22.8	−8.8	−6.8	−0.4	−1.4*
Croatia	5.2	3.6	9.4	81.1	159.3	2.6*	5.9*	12.4	9.0	19.3	56.0	112.9	1.9*	8.1*
Greece	1.5	17.8	20.9	1324.8	17.5	12.0*	2.0	6.1	15.9	20.4	232.9	28.6	4.1	1.1
Italy	10.6	10.2	13.8	30.3	35.2	1.0	2.2	18.6	18.9	21.3	14.9	13.1	1.2	1.0
Portugal	11.8	9.7	14.9	26.4	53.0	1.4*	3.1	13.2	17.0	13.4	2.2	−21.0	0.0	−2.2
Slovenia	6.4	9.3	15.8	148.4	70.4	2.7*	5.3	18.8	10.5	24.8	32.5	136.2	2.0*	2.1
Spain	32.5	29.3	21.2	−34.6	−27.5	−1.8*	−3.5*	44.3	38.9	27.8	−37.2	−28.4	−1.9*	−4.0*
Western Europe (*n* = 6)	19.1	19.0	16.6	−12.7	−12.6	−0.8	−1.1*	25.5	21.0	19.0	−25.4	−9.6	−1.3*	−1.2*
Austria	8.5	6.4	9.6	12.1	49.5	−0.3	0.7	17.5	8.5	14.8	−15.8	73.7	−0.5	2.5
Belgium	9.5	19.4	16.7	76.3	−14.0	2.5*	−1.9	15.3	23.1	18.7	22.3	−19.1	1.0	−2.3*
France	23.8	29.1	20.4	−14.4	−29.9	−1.7	−3.1*	28.7	29.4	21.5	−25.2	−27.0	−1.3*	−3.6*
Germany	16.4	9.9	13.5	−17.7	35.6	−1.2*	3.4*	23.2	12.5	15.7	−32.5	25.3	−1.7*	2.7*
Netherlands	24.5	25.3	19.0	−22.5	−25.0	−1.4*	−2.9*	37.7	37.1	25.2	−33.2	−32.0	−2.1*	−3.7*
Switzerland	24.5	24.5	19.7	−19.4	−19.6	−0.0	−3.2*	27.7	33.4	29.1	4.9	−13.0	−0.2	−2.7*
Caribbean (*n* = 4)	11.7	17.6	18.5	58.0	5.1	1.9*	2.1	22.7	28.3	31.1	37.1	9.7	1.1*	1.2
Cuba	13.5	18.8	23.4	73.8	24.2	2.7*	3.8	28.1	32.3	38.7	37.6	19.9	0.8	3.0
Dominican Republic	8.4	5.4	3.0	−64.2	−44.2	−2.5	−5.6	9.3	8.6	7.1	−23.6	−17.9	0.3	−0.8
Puerto Rico	5.7	25.8	22.7	295.5	−12.0	5.7*	1.2	13.2	33.5	35.0	164.5	4.6	3.5*	1.2
Trinidad and Tobago	54.6	43.4	44.7	−18.1	3.0	−1.3	0.7	81.8	65.9	75.0	−8.3	13.8	−0.1	−0.2
Central America (*n* = 4)	47.3	26.9	33.9	−28.4	26.1	−1.1	4.1*	61.8	45.1	52.7	−14.8	16.9	−0.4	2.9*
Costa Rica	15.4	19.7	31.6	104.9	60.3	2.7	6.4*	47.9	35.7	52.5	9.6	47.1	0.2	5.2*
Guatemala	68.7	28.2	18.8	−72.6	−33.1	−5.6*	−4.6*	48.0	21.4	26.2	−45.3	22.8	−2.5	1.0
Mexico	46.6	27.7	36.4	−21.9	31.5	−0.7	4.7*	63.7	48.2	56.4	−11.5	17.0	−0.2	3.0*
Panama	42.0	5.7	5.9	−85.9	3.2	−4.0*	4.3	37.1	25.2	16.0	−57.0	−36.7	−1.8*	−0.2
South America (*n* = 8)	12.8	16.6	19.7	53.9	19.2	1.6*	2.1*	22.2	26.1	31.1	40.5	19.4	1.3*	2.1*
Argentina	11.0	8.2	13.0	18.2	58.7	0.6	3.9*	18.5	17.7	22.8	22.7	28.2	0.5	2.6*
Brazil	9.3	18.0	20.4	119.7	13.1	3.3*	1.6*	16.6	23.0	28.6	72.8	24.7	2.8*	2.6*
Chile	9.1	17.0	15.1	65.6	−11.3	1.6*	−1.7	31.5	37.2	31.4	−0.3	−15.6	−0.2	−2.0*
Colombia	25.4	27.8	43.0	69.1	54.4	1.7	4.7*	35.2	45.3	69.3	96.8	53.2	2.8*	4.8*
Ecuador	31.1	7.4	11.6	−62.6	56.3	−3.5	3.4	55.2	20.1	25.2	−54.4	25.2	−2.6	5.8*
Peru	6.0	12.9	8.9	47.4	−31.6	−2.1*	−4.4	16.5	22.0	16.5	0.3	−24.9	−1.5	−4.1
Uruguay	11.0	18.9	24.7	124.5	31.2	3.0*	3.9	18.3	31.7	38.5	110.7	21.6	2.9*	3.0*
Venezuela	22.9	16.5	10.3	−54.8	−37.5	−2.8*	−4.6*	38.1	42.8	30.4	−20.1	−28.9	−1.0	−3.7*
North America (*n* = 2)	14.0	22.6	20.0	42.4	−11.4	1.3*	−1.5*	24.1	38.1	30.2	25.1	−20.7	0.9*	−2.6*
Canada	18.2	20.9	18.7	2.5	−10.7	0.2	−1.3*	26.4	34.1	28.5	7.9	−16.3	0.1	−2.9*
USA	13.7	22.8	20.1	47.5	−11.5	1.4*	−1.6*	24.0	38.6	30.4	26.5	−21.3	0.9*	−2.6*
Oceania (*n* = 2)	25.2	24.0	21.0	−16.4	−12.3	−1.1	−1.9*	33.1	34.3	31.1	−6.0	−9.2	−0.2	−1.3*
Australia	24.3	22.6	20.3	−16.3	−10.1	−0.6*	−1.8*	31.2	32.6	29.7	−4.7	−8.8	−0.3*	−1.2*
New Zealand	29.3	30.9	24.8	−15.4	−19.8	0.0	−2.3*	42.4	42.6	38.5	−9.0	−9.6	0.4	−1.9*

*Average annual percent change is statistically significantly different from zero (*P* < 0.05)

For women, the mean MSK mortality rate declined from 26.6 deaths per million person-years in 1986 to 25.1 in 2011, representing a 5.6% reduction (ranging from a 76.5% reduction in Singapore to a 233% increase in Greece with a median of 9.0% reduction). Mortality decreases of 25% or more were observed in 19 countries, while corresponding increase were seen in 14 countries. The absolute change ranged from 30.5 less MSK deaths per million person-years in England & Wales to about 34 more deaths in Colombia between 1986 and 2011. Across regions, the highest MSK mortality decline was seen in Northern Europe (41.1% reduction) and the highest increase in South America (40.5% increase). Age-specific MSK mortality rates declined in most age groups with the highest decline and increase for 0–9 years and 25–29 years age groups, respectively.

In 39 out of 58 countries women experienced more favorable changes (i.e., either more reductions or less increases) compared with men. In addition, the absolute differences in MSK mortality between women and men declined from 9.4 deaths per million person-years in 1986 to 6.9 deaths in 2011. Corresponding women to men rate ratio (95% CI) declined from 1.54 (1.51 to 1.58) to 1.38 (1.36 to 1.40) during the same period. Moreover, in 15 out of 18 age groups, women experienced more favorable changes in age-specific MSK mortality rates compared with men.

### Joinpoint Regression analysis

**Fig. 3:**
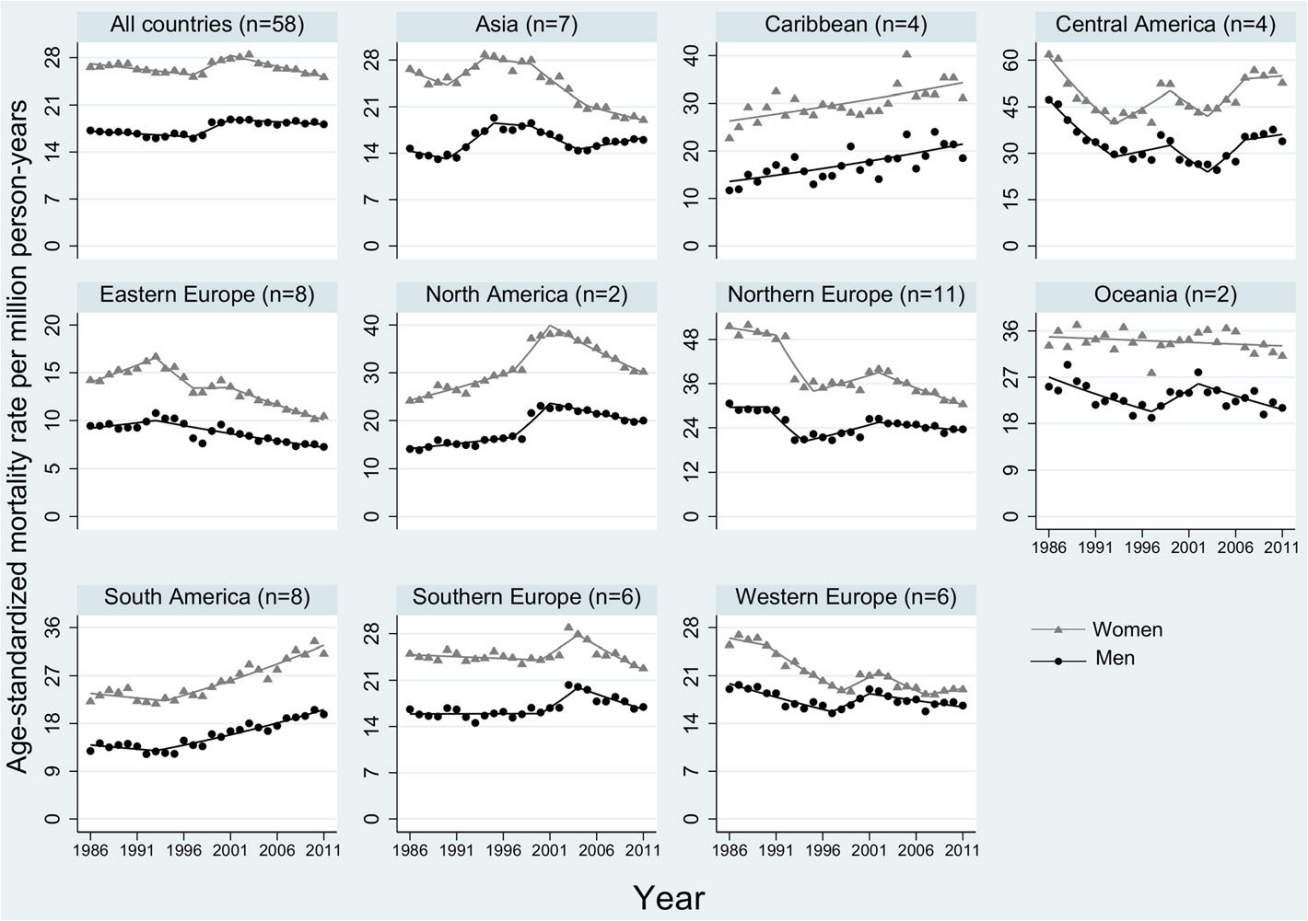
Temporal trend in age-standardized musculoskeletal mortality rates across regions, 1986–2011. *Footnote: Symbols display observed values and solid lines show fitted values from joinpoint regression*

The pooled MSK mortality rate decreased between 1986 and 1997, then increased during 1997–2001 and again decreased thereafter in both sexes (with lower rate of increase and higher rate of decrease for women compared with men, Table 1 and Fig. 3). Moreover, while the trend for whole period revealed statistically non-significant annual changes during 1986–2011 (0.2% annual increase and 0.3% annual decrease for men and women, respectively), the recent trend (2001–2011) showed a statistically significant reduction of 0.3% and 1.2% per year among men and women, respectively. However, there were substantial between-country variations in temporal trend (Additional file 4: Figure S3 and Additional file 1: Table S3). During 1986–2011, the highest annual reduction was observed in Romania and highest annual increase in Greece for both sexes. On the other hand, during the most recent decade the highest reduction was observed among women in Republic of Moldova (9.9% annual reduction) and the highest increase among men in Czech Republic (15.0% annual increase).

### Between-country disparities

**Fig. 4:**
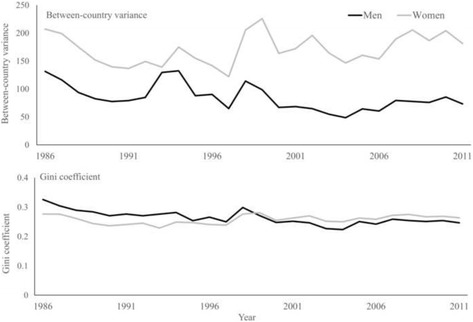
Changes in absolute and relative between-country disparities in musculoskeletal disorders mortality rate over time, stratified by sex

The between-country variance in MSK mortality declined from 131.4 and 207.2 death per million person-years in 1986 to 73.3 and 181.6 in 2011 among men and women, respectively (Fig. 4). In all study years, the magnitude of the between-country variance was higher among women compared with men. The relative between-country disparity measured by Gini coefficient declined from 0.33 and 0.28 in 1986 to 0.25 and 0.26 in 2011 among men and women, respectively. While during 1986–1999, the magnitude of relative between-country disparities were higher among men compared with women, there were lower disparities among men thereafter.

### Decomposition analysis of the number of MSK deaths

**Fig. 5:**
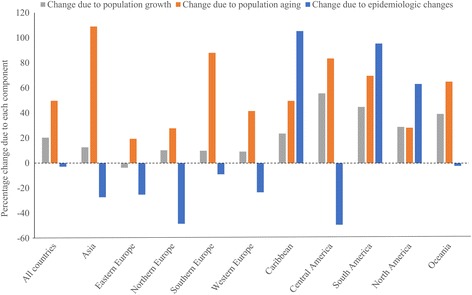
Percent change in number of musculoskeletal disorders deaths due to population growth, population aging, and epidemiologic changes, 1986–2011

In 2011, the number of MSK deaths increased by 67% compared to 1986 and this was mainly due to population aging (Fig. 5). In six regions the number of MSK deaths increased due to population growth and aging and declined due to epidemiologic changes, but only in Northern Europe this led to an actual decline in the number of MSK deaths. In Eastern Europe the combination of increase in the number of MSK deaths due to aging population and declines due to population growth and epidemiologic changes translated into a 9% reduction in the number of MSK deaths. In Caribbean, South America, and North America number of MSK deaths due to all three components increased.

Across countries, the number of MSK deaths due to population growth, aging and epidemiologic changes increased in 26 countries (Additional file 1: Table S4). In twenty countries despite the declines due to population growth (4 countries) or epidemiologic change (16 countries), the total number of MSK deaths increased between 1986 and 2011. A total of 12 countries presented reductions in the total number of MSK deaths which caused due to either epidemiologic change (Singapore, Poland, Finland, England & Wales, Guatemala, and Panama) or population growth (Estonia) or both (Republic of Moldova, Romania, Russian Federation, Ukraine, and Lithuania).

## Discussion

From 1986 to 2011, on average across all countries, the total number of MSK deaths and its proportion from all-cause deaths increased in both sexes and this increase was mainly due to population aging. In addition, the age-standardized MSK mortality rate increased by 5.3% in men and declined by 5.6% in women during the same period. Although more women died from MSK compared with men, women experienced more favorable changes during the study period. While there were substantial relative and absolute disparities in MSK mortality between countries, these disparities have declined over time with more profound reduction among men.

The total number of MSK deaths increased due to population growth and aging without a significant reduction due to epidemiologic changes. Across regions while MSK deaths due to epidemiologic changes declined in most regions, these reductions led to actual reductions in MSK deaths only in Eastern and Northern Europe. These findings highlight importance of these demographic forces particularly aging in MSK deaths which should be taken into account by health policy-makers. It should be noted that changes in ICD revision and quality of vital registrations are included in epidemiologic changes and might partly offset reductions due to other epidemiologic changes including improvement in MSK management.

We found substantial variations between regions/countries not only in level of MSK deaths but also in temporal changes of MSK deaths. For example, MSK deaths increased in Southern Europe, Caribbean, Central America, and South America over recent decade. Differences between regions/countries in availability and access to treatments, socioeconomic status, prevalence of MSK risk factors including obesity, epidemiology of disease, quality of vital registration, and cause of death certification including transition from paper to electronic certification and from manual to automated coding system might partially explain these disparities. Moreover, previous studies reported racial disparities in prevalence of musculoskeletal disorders and access to treatment which might partially explain the observed disparities in our study [20–22]. Furthermore, although both relative and absolute between-country disparities have declined over time still substantial disparities are present. This is particularly of concern among women where compared with men the relative inequality slightly declined between 1986 and 2011 (5% reduction among women vs. 24% reduction among men). It should be noted that the observed disparity in recent decade cannot be attributed to between-country differences in ICD revision since many countries (46 out of 58 countries) were applying the same ICD revision during 2001–2011. Further analyses are required to investigate these disparities in more details.

The observed increases in pooled MSK mortality rates during 1997–2001 are possibly due to the introduction of ICD-10 coding system in many countries (most countries introduced ICD-10 between 1995 and 2001) considering that a ICD-10 to ICD-9 comparability ratio greater than 1 have been reported for MSK deaths [23–25]. While not case for all countries but we observed a jump in MSK mortality rates in many countries in early years of introducing ICD-10 revision. This suggests that taking the impact of ICD-10 introduction into account would be associated with a steady reduction in pooled MSK mortality rates over the whole study period. Global focus on MSK particularly since endorsement of the Bone and Joint Decade 2000–2010 [26] by the United Nations and the WHO might partly explain observed reduction in MSK mortality rate. Moreover, new interventions particularly the biologic agents for RA, emergence of new imaging technologies, and advances in the rapid surgical procedures substantially changed the clinical management of patients and might partially explain the observed declining trend in MSK mortality rates [2]. This decline in mortality rate alongside population aging imply potential increases in burden of MSK in coming years and health policy-makers should be aware of this. Several strategies has been suggested in response to this expected increase in burden of MSK including raising patients’ awareness about importance of a healthy life style, raising awareness of health professionals through providing adequate training in MSK, early diagnosis and treatment of MSK, improving access to MSK therapies including rehabilitation, implementation of integrated and patient-centred multi-disciplinary models of care, and delivery of primary prevention initiatives at a population level [6, 27–29].

Almost in all countries women had higher MSK mortality rate compared with men. Several explanations have been suggested for this sex disparity including higher prevalence of MSK among women, sex differences in biological and hormonal factors, in severity and remission from MSK, in access and responses to treatments, and in susceptibility developing other complications such as cardiovascular disease [30–35]. While sex disparity in MSK mortality declined over the study period, substantial effort and resources are required to close the observed gap. For example, if we naively assume that men will continue to have an annual reduction of 0.3% observed in 2001–2011 until 2025, then the annual reduction among women should be doubled (from 1.2 to 2.6%) to close sex disparity in MSK mortality rate by 2025.

In interpreting the results of the current study, several limitations should be considered. First, MSK are underreported as underlying cause of deaths on death certificates and the degree of underreporting might vary between time and space that could bias our findings. Similarly, there are between country/time variations in presence of errors or incompleteness in death certificates which might bias our results. Second, changes in the death certification, coding process over time (e.g., a transition from paper to electronic certification, from manual to automated coding system, from ICD-9 to ICD-10) might bias the results of mortality trends. For example, considering a ICD-10 to ICD-9 comparability ratio above 1, our estimates for countries with declining trends are possibly an underestimation of the magnitude of the true trend, and for countries with increasing trend our estimates could be either an overestimation of the magnitude of the true trend or a bias in direction of change. However, without knowing the country-specific comparability ratios it is hard to quantify the size of biases in our estimations. Furthermore, it should be noted that this differences in ICD revision could not account for the observed disparities in most recent decade since most countries were applying ICD-10 revision over this period. Third, due to lack of data we were not able to investigate mortality of MSK subcategories. For example, only 29 (mostly European countries) out of 58 countries had the required data on mortality of RA over the study period (in these countries RA constituted 22% of all MSK deaths). Fourth, the countries included in our study were mainly upper-middle and high income countries with a reliable vital registration system and available data on WHO mortality database and therefore our results might not be generalizable to lower-middle and low income countries. Fifth, the small number of deaths in some countries might have limited the power of our study to detect significant joinpoints over the study period. In addition, direct age-standardization method used in the study is sensitive to small number of events and the results for countries with low number of events should be interpreted with caution. It also should be noted that the WHO standard population was developed in 2001 and might not reflect demographic changes occurred since 2001. Furthermore, the disparity measures applied in the study are sensitive to outliers and cannot capture socioeconomic gradient in MSK mortality. Sixth, this is a descriptive aggregate-level study and no causal inferences should be made from the findings. Despite these limitations, to our best knowledge, this is the first study to investigate temporal trend and between-country disparities in MSK mortality across a large number of countries. The results of the current study might provide useful insights on epidemiological status of MSK and can be used by policy-makers in planning MSK management at both national and global level.

## Conclusion

The total number of MSK deaths and its proportion from all-cause deaths increased between 1986 and 2011 and this was mainly due to population aging. On the other hand, taking the potential impact of ICD-10 revision into account, the pooled mean age-standardized MSK mortality rate declined over the study period with a more favorable reduction among women. The highest MSK mortality rates were observed in Central America and the lowest in Eastern Europe. Between 1986 and 2011, the highest reduction in MSK mortality rate was observed in women in Northern Europe and the highest increase in men in Caribbean. Increases in MSK mortality rates in Southern Europe, Caribbean, Central America, and South America during most recent decade require further actions. Further investigations are required to explain substantial absolute and relative disparities in MSK mortality rate and its temporal trend between sexes, countries, and regions.

## Additional files

Additional file 1: Excel file including all supplementary tables. (XLSX 41 kb)
Additional file 2:
**Figure S1**. Proportion of musculoskeletal disorders deaths from all-cause deaths 1986–2011, stratified by sex. (TIFF 578 kb)
Additional file 3:
**Figure S2**. The mean age-standardized musculoskeletal disorders mortality rates per million person-years by sex and region, 1986–2011. (TIFF 483 kb)
Additional file 4:
**Figure S3**. Temporal trend in age-standardized musculoskeletal mortality rates by sex and country, 1986–2011. *Footnote: Symbols display observed values and solid lines show fitted values from joinpoint regression. Vertical line shows the year of the introduction of ICD-10 revision*. (PDF 156 kb)

## Abbreviations

AAPC: Average annual percent change
APC: Annual percent change
BCV: Between-country variance
CI: Confidence interval
ICD: International Classification of Diseases
MSK: Musculoskeletal disorders
WHO: World Health Organization
