# Supplementary figures and images for: Musculoskeletal disorders as underlying cause of death in 58 countries, 1986–2011: trend analysis of WHO mortality database
Source: BMC Musculoskelet Disord. 2017 Feb 2;18:62. doi: 10.1186/s12891-017-1428-1 (PMC5290674; doi:10.1186/s12891-017-1428-1)

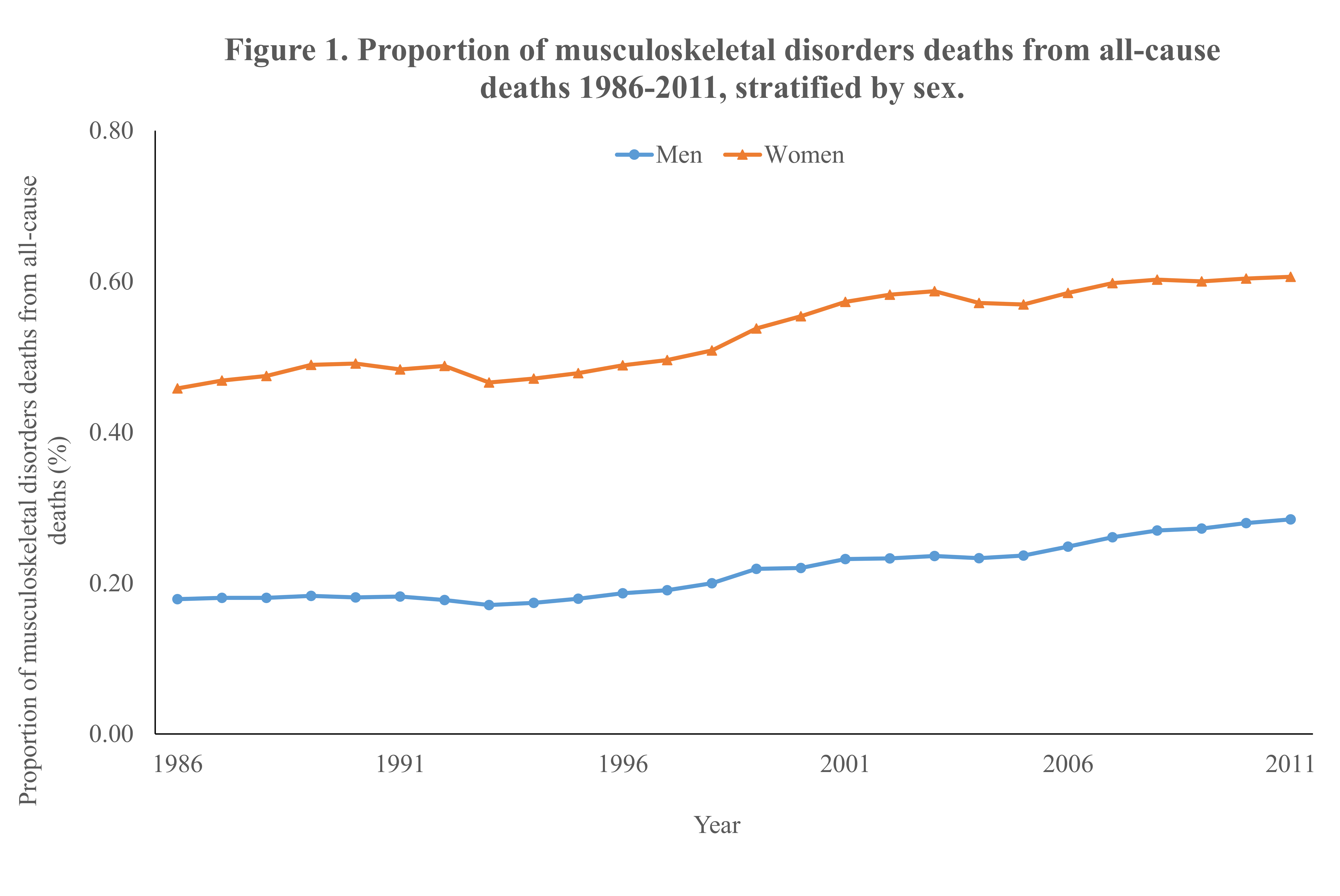

Supplement: Additional file 2: — Figure S1. Proportion of musculoskeletal disorders deaths from all-cause deaths 1986–2011, stratified by sex. (TIFF 578 kb) [file 12891_2017_1428_MOESM2_ESM.tiff]

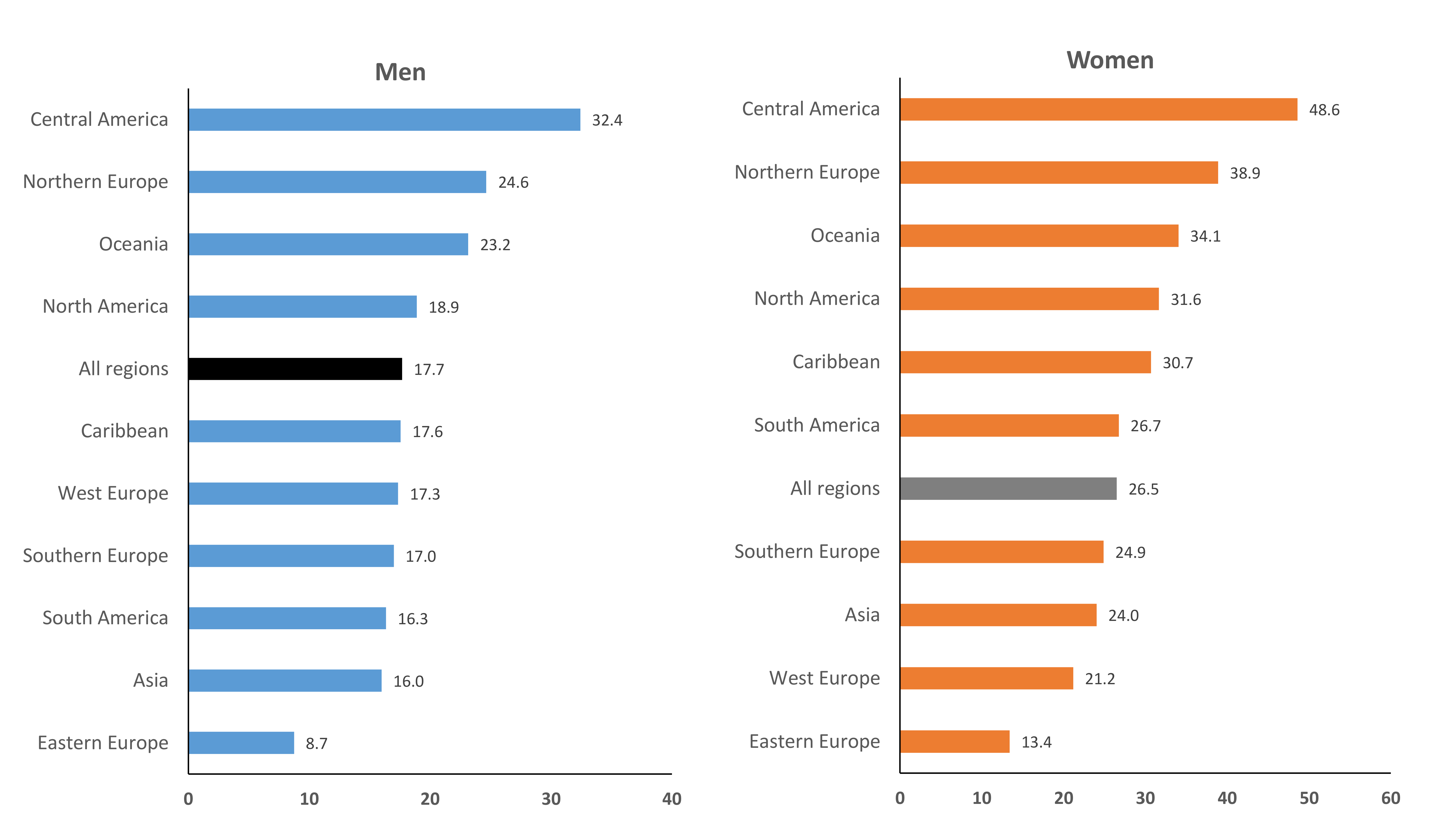

Supplement: Additional file 3: — Figure S2. The mean age-standardized musculoskeletal disorders mortality rates per million person-years by sex and region, 1986–2011. (TIFF 483 kb) [file 12891_2017_1428_MOESM3_ESM.tiff]
