# Supplementary material for: Musculoskeletal disorders as underlying cause of death in 58 countries, 1986–2011: trend analysis of WHO mortality database
Source: BMC Musculoskelet Disord. 2017 Feb 2;18:62. doi: 10.1186/s12891-017-1428-1 (PMC5290674; doi:10.1186/s12891-017-1428-1)
Supplement: Additional file 4: — Figure S3. Temporal trend in age-standardized musculoskeletal mortality rates by sex and country, 1986–2011. Footnote: Symbols display observed values and solid lines show fitted values from joinpoint regression. Vertical line shows the year of the introduction of ICD-10 revision. (PDF 156 kb) [file 12891_2017_1428_MOESM4_ESM.pdf]

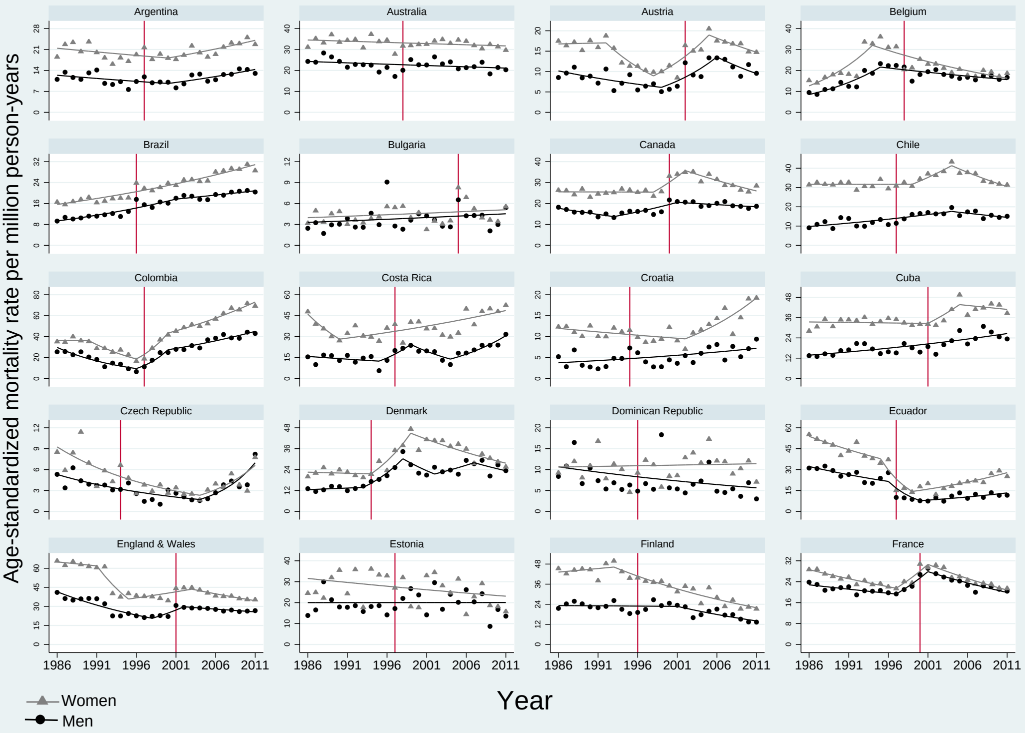

Argentina
Australia
Austria
Belgium
Brazil
Bulgaria
Canada
Chile
Colombia
Costa Rica
Croatia
Cuba
Czech Republic
Denmark
Dominican Republic
Ecuador
England & Wales
Estonia
Finland
France
Age-standardized mortality rate per million person-years
Year
1986 1991 1996 2001 2006 2011
Women
Men

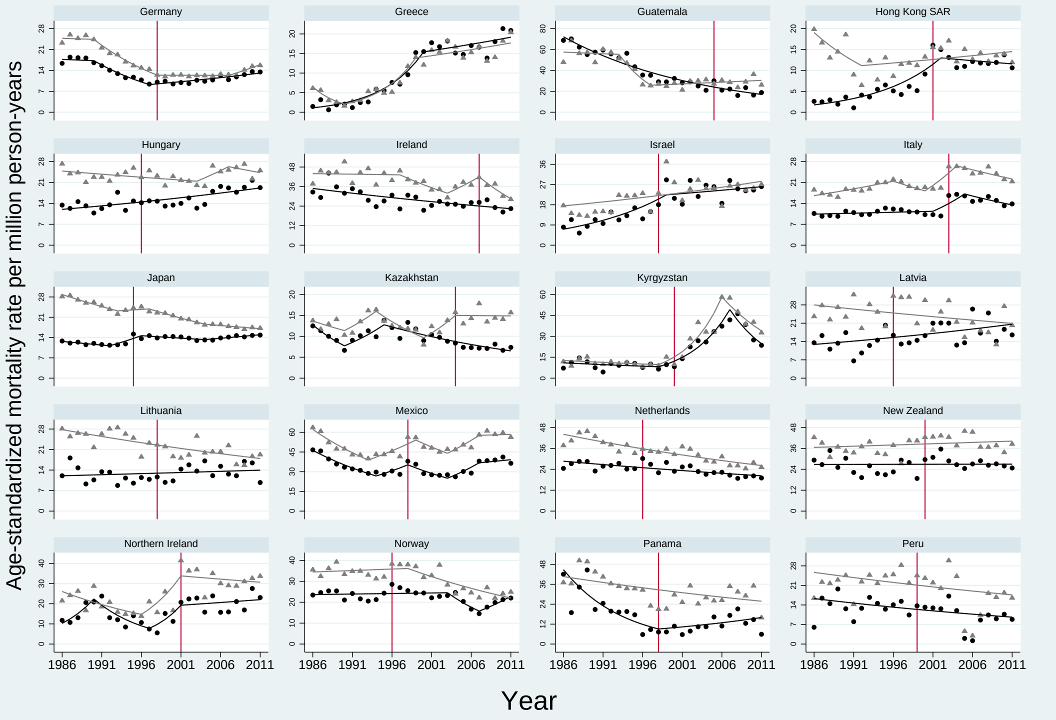

Age-standardized mortality rate per million person-years
Year
Germany
Greece
Guatemala
Hong Kong SAR
Hungary
Ireland
Israel
Italy
Japan
Kazakhstan
Kyrgyzstan
Latvia
Lithuania
Mexico
Netherlands
New Zealand
Northern Ireland
Norway
Panama
Peru
1986 1991 1996 2001 2006 2011

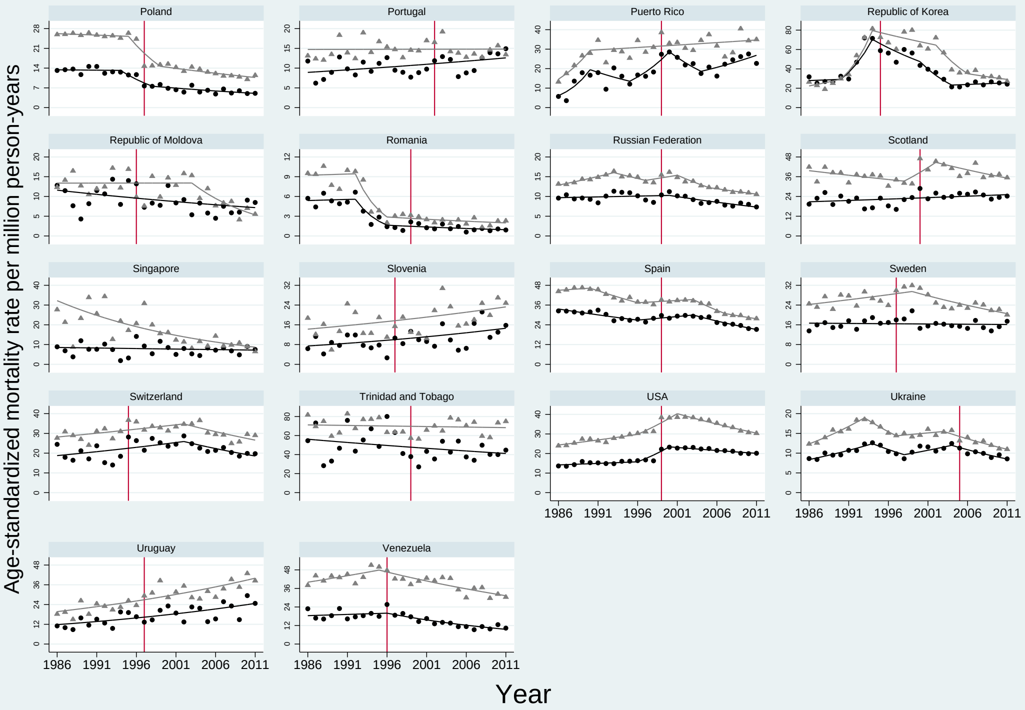
Poland
Portugal
Puerto Rico
Republic of Korea
Republic of Moldova
Romania
Russian Federation
Scotland
Singapore
Slovenia
Spain
Sweden
Switzerland
Trinidad and Tobago
USA
Ukraine
Uruguay
Venezuela
Age-standardized mortality rate per million person-years
Year
1986 1991 1996 2001 2006 2011
